# Use of reusable menstrual management materials and associated factors among women of reproductive age in Ghana: analysis of the 2017/18 Multiple Indicator Cluster Survey

**DOI:** 10.1186/s12905-022-01670-9

**Published:** 2022-03-26

**Authors:** Emmanuel Anongeba Anaba, Emilia Asuquo Udofia, Adom Manu, Anita Anima Daniels, Richmond Aryeetey

**Affiliations:** 1 Department of Population, Family and Reproductive Health, School of Public Health, College of Health Sciences, University of Ghana, Accra, Ghana; 2 Department of Community Health, College of Health Sciences, University of Ghana Medical School, University of Ghana, Accra, Ghana; 3 Department of Public Administration and Health Services Management, Business School, College of Humanities, University of Ghana, Accra, Ghana

**Keywords:** Reusable menstrual materials, Women in reproductive age, Ghana, Sanitary pad

## Abstract

**Background:**

The use of unsafe materials to collect menstrual blood predisposes women and girls to infections. There is a paucity of literature on the utilization of reusable menstrual materials in sub-Saharan Africa. This study examined factors associated with the use of reusable menstrual management materials among women of reproductive age in Ghana. Findings from this study can inform menstrual health programmes and reproductive health policy to address menstrual hygiene and specific areas of emphasis.

**Methods:**

We analysed secondary data from the 2017/18 Ghana Multiple Indicator Cluster Survey. Descriptive statistics were employed to compute frequencies and percentages, while Chi-square and complex sample Binomial Logistic Regression was conducted to identify factors associated with the use of reusable menstrual materials.

**Results:**

Half (52%) of the respondents were below 30 years old; mean (± sd) = 30.7(9.0). Thirteen percent used reusable materials to collect menstrual blood during their last period. Women aged 45–49 years (AOR = 5.34; 95% CI 3.47–8.19) were 5 times more likely to manage menstruation with reusable materials compared with those aged 15–19 years (*p* < 0.05). Women classified in the middle wealth quintile (AOR = 0.66; 95% CI 0.50–0.88) were 34% less likely to use reusable materials to collect menstrual blood compared with women in the poorest wealth quintile (*p* < 0.05). Also, women who were exposed to television (AOR = 0.78; 95% CI 0.61–0.99) had less odds of using reusable materials compared with women who were not exposed to television (*p* < 0.05).

**Conclusion:**

This study showed that the use of reusable menstrual materials was influenced by socio-demographic factors, economic factors and exposure to mass media. Therefore, policies and programmes aimed at promoting menstrual health should focus on less privileged women. The mass media presents an opportunity for communicating menstrual hygiene.

## Background

Menstruation is a normal biological process that marks the beginning of the reproductive age. Globally, 1.8 billion women of reproductive age menstruate every month [1]. Promoting optimal hygiene during menstruation is crucial for preventing menstruation-related infections. Unfortunately, about 500 million women and girls lack access to menstrual hygiene facilities, including safe and clean menstrual management materials (MMMs) [2]. There are two main types of MMMs: disposable materials (including tampon and disposable sanitary pad) and reusable materials (including reusable pad, cloth, and menstrual cup) [3]. Disposable menstrual materials are mostly considered clean by women. Reusable materials can also be classified as clean if they are cleaned with soap, dried in sunlight, and stored in a clean place [4, 5]. However, effective management of reusable menstrual materials, especially reusable cloth/pads, remains a challenge for many women [6]. Reusable pads/cloths are often not appropriately cleaned and stored under hygienic conditions [7]. Mostly, menstruators dry them indoors or keep them in the toilet room, due to socio-cultural beliefs and norms surrounding menstruation [8]. Also, the cleaning of reusable menstrual materials requires water and soap, which may not always be available [9]. These challenges may predispose users to reproductive tract infections and complications.

Evidence on the safety of reusable menstrual materials is mixed. Some studies have reported that reusable menstrual materials are associated with reproductive tract infections [10–12]. However, other studies have demonstrated that reusable menstrual management materials are safe, reliable and accepted by menstruators [13, 14]. Reusable menstrual materials are more cost-effective and environmentally friendly compared with single-used pads [7]. Women and adolescents in low-income countries prefer reusable pads because they are culturally acceptable, user-friendly, affordable, clean, effective, and accessible [11, 15, 16]. There is a preference for reusable pads, especially among women in rural settings [5]; however, young women preferred disposable sanitary products. Adolescent girls felt that it was old fashioned to collect menstrual blood with reusable materials [9].

In Ghana, adolescent girls use reusable materials to manage menstruation [17]. A considerable proportion (including 44% and 54.2%) of schoolgirls in northern Ghana use reusable cloth pads to manage menstruation due to lack of access to menstrual hygiene facilities, including safe and clean sanitary products. Many girls in rural and poor households lack access to disposable sanitary pads and resort to unsafe materials to collect menstrual blood [17]. Adolescent girls use old cloth, panty liners, pieces of mattress or cotton to collect menstrual blood [18]. Girls lack access to disposable pads due to financial constraints, unavailability and stigma. Most girls do not have income for purchasing disposable sanitary pads [17]. In addition, menstruators dry reusable menstrual materials indoors or under the shade [19]. Moreover, awareness and uptake of menstrual cups are very low in Ghana [18]. This suggests that a majority of women who use reusable menstrual materials may be using reusable cloth/pads.

There is limited empirical evidence for the correlates of using reusable menstrual materials. Prior studies in Ghana focused on assessing knowledge, attitudes and menstrual hygiene practices among school girls [17, 18]. For instance, [18] revealed that the majority of school girls in northern Ghana involved in unhygienic menstrual practices, such as using unsafe materials (i.e. old cloth, pieces of mattress and cotton) to collect menstrual blood. The correlates of good menstrual hygiene practices included age of respondent, staying with both parents, access to funds and adequate knowledge about menstruation. In addition, [17] demonstrated that maternal education, late adolescence (15–19 years) and exposure to radio and television were protective factors against poor menstrual knowledge among school girls in northern Ghana. None of the existing studies explored the correlates of reusable menstrual materials among women of reproductive age. Further, the use of menstrual materials is influenced by context-specific factors, such as cultural beliefs, availability and socio-economic factors [20, 21]. Hence generalizing findings from other countries would be inappropriate. This is the maiden study in Ghana to examine the use of reusable menstrual materials among women of reproductive age using nationally representative data. Findings from this study can inform menstrual health programmes and reproductive health policy to address menstrual hygiene and specific areas of emphasis. The objective of this study was to identify factors associated with the use of reusable menstrual materials among women of reproductive age in Ghana.

## Methods

### Study location, design and source of data

Ghana is one of the countries in West Africa and shares boundaries with Togo to the east, Cote d’Ivoire to the west, Burkina Faso to the north and the Gulf of Guinea to the south. Ghana has a total population of about 30.8 million with the majority being females (50.7%) [22]. Women and girls in Ghana face several menstruation-related challenges, including lack of access to safe menstrual materials, water, sanitation and hygiene facilities, hence they are predisposed to infections and school absenteeism [18, 23]. This study analysed secondary data from the 2017/18 Multiple Indicator Cluster Survey (MICS). The 2017/2018 MICS was the sixth round of MICS in Ghana, nationally representative and the most recent. Unlike previous MICS, this survey employed Computer-Assisted Personal Interviewing (CAPI) to collect data from the participants. Participants were recruited across the previous ten administrative regions in the country, employing a two-stage sampling technique. The first stage of the recruitment process involved the selection of 660 clusters from the 2010 Population and Housing Census sampling list proportional to size. Secondly, 13,202 households (including 6163 from urban areas and 6864 from rural areas) were selected from the clusters using a systematic random sampling technique. This study focused on women of reproductive age, hence we analysed data from the women file. After dropping missing values and adjusting for the complex nature of the survey, we obtained a weighted sample size of 10,861 women aged 15–49 years. The 2017/18 MICS report indicated that verbal consent was obtained from all participants before questionnaires were administered. Informed consent was obtained from parents or legal guardians of participants below 18 years (minors). Moreover, participants were assured of voluntary participation, confidentiality, anonymity of information, and free will to withdraw from the interview at any point. Data analysed in this study was obtained from UNICEF through a formal request.

### Measurement

The dependent variable in this study was the use of reusable menstrual management materials. This was a direct follow-up question to materials used during the last menstruation (*were the materials reusable?).* This item was originally coded as (Yes = 1, No = 2, and don’t know = 8). Before the analysis, the outcome was re-coded as ‘Yes’ = 1 and ‘No’ = 0. Responses indicating ‘don’t know’ and ‘non-response’ were deleted from the dataset. The independent variables identified were socio-demographic characteristics, disability status, and exposure to the mass media. The following socio-demographic variables were included in analyses: age of respondent, educational status, household wealth index, marital status, type of place of residence, and region. Disability status was coded as ‘has functional difficulties’ = 1 and ‘has no functional difficulties’ = 2. Exposure to mass media was assessed using three items: frequency of reading a newspaper, listening to a radio, and watching television. Details about the coding of the independent variables are provided elsewhere [24].

### Statistical analysis

We employed univariate, bivariate, and multivariable analyses. At the univariate level, descriptive statistics were computed using frequency and percentages and summarized in tables. At the bivariate level, the Chi-square test was used to examine the association between the dependent and independent variables. Binomial Logistic Regression was used to identify significant predictors of the outcome variable. All the statistical tests were embedded in Stata/SE version 16. Further, we adjusted for the complex nature of the survey (clustering, stratification and sampling weight) by employing the ‘*svy*’ Stata command. All statistical significance was reported at the 0.05 significance level, while the odds ratios and their 95% confidence intervals were used to examine the strength of association.

## Results

### Descriptive statistics

The results showed that 21% of the participants were aged 15–19 years, 15% were between the ages of 30–34 years and 8% were between the ages of 45–49 years. The mean age was 30.7 years and a standard deviation of 9.0. (Table 1). Exactly 41% of the participants had completed junior high education, while 17% had pre-primary/no education. About 16% of the women were classified in the poorest wealth quintile and 24% in the richest wealth quintile. The majority (55%) of the participants were currently married, and 36% had never married. Fifty-two per cent of the participants resided in urban areas, 25% were from the Ashanti Region, and 9% had disabilities. The majority (89%) of the women did not read a newspaper, 31% did not listen to a radio, and 46% watched television almost every week. The prevalence of using reusable menstrual materials was 13% and 87% used disposable menstrual materials.

**Table 1:** Participants’ characteristics

Characteristic	Frequency	Percentage
*Age (years)*		
15–19	2697	21
20–24	2023	16
25–29	1892	15
30–34	1836	15
35–39	1678	13
40–44	1462	12
45–49	993	8
*Education level completed*		
Pre-primary/no education	2156	17
Primary	2092	17
Junior high	5158	41
Senior high	2402	19
Higher	774	6
*Wealth index categories*		
Poorest	1973	16
Second	2287	18
Middle	2551	20
Fourth	2713	22
Richest	3058	24
*Marital status*		
Currently married	6884	55
Formerly married	1190	9
Never married	4508	36
*Area of residence*		
Urban	6508	52
Rural	6074	48
*Region*		
Western	1275	10
Central	1255	10
Greater Accra	1677	13
Volta	905	7
Eastern	1520	12
Ashanti	3101	25
Brong-Ahafo	1138	9
Northern	1086	9
Upper East	359	3
Upper West	265	2
*Disability status*		
Has functional difficulty	974	9
Has no functional difficulty	9887	91
*Frequency of reading newspaper*		
Not at all	11,000	89
Less than once a week	796	6
At least once a week	559	4
Almost every week	166	1
*Frequency of listening to a radio*		
Not at all	3918	31
Less than once a week	2213	18
At least once a week	2672	21
Almost every week	3779	30
*Frequency of watching television*		
Not at all	3173	25
Less than once a week	1478	12
At least once a week	2187	17
Almost every week	5744	46
*Reusable menstrual material*		
Yes	1614	13
No	10,907	87

### Association between participants’ characteristics and usage of reusable materials

A higher proportion (29%) of women aged 45–49 years used reusable menstrual materials compared with women aged 15–19 years (6%) (*p* < 0.05). Also, the use of reusable materials was significantly associated with educational status (*p* < 0.05). Thirty-eight per cent of the women with no education used reusable materials, while only 2% of the women with higher education used reusable materials. A considerable proportion of women in the poorest (29%) wealth quintile managed menstruation with reusable materials. Significant associations were found between marital status, disability, and the use of reusable materials (*p* < 0.05). Four in ten women in the Northern region used reusable materials to collect menstrual blood. A substantial proportion of women who had less exposure to mass media, including newspaper (14%), radio (18%) and television (24%), preferred reusable materials (*p* < 0.05) (Table 2).

**Table 2:** Cross-tabulation of participant characteristics and use of reusable pads

Characteristic	Menstrual material reusable			
	Total (n)	Yes (%)	No (%)	Chi-square
*Age groups (years)*				
15–19	2697	6	94	62.07*
20–24	2023	6	94	
25–29	1892	9	91	
30–34	1836	12	88	
35–39	1678	17	83	
40–44	1462	24	76	
45–49	993	29	71	
*Education completed*				
Pre-primary/no education	2156	38	62	204.94*
Primary	2092	16	84	
Junior high	5158	7	93	
Senior high	2402	3	97	
Higher	774	2	98	
*Wealth index*				
Poorest	1973	29	71	113.92*
Second	2287	21	79	
Middle	2551	13	87	
Fourth	2713	5	95	
Richest	3058	3	97	
*Marital status*				
Currently married	6884	18	82	97.22*
Formerly married	1190	15	85	
Never married	4508	5	95	
*Area of residence*				
Urban	6508	8	92	93.84*
Rural	6074	18	82	
*Region*				
Western	1275	13	87	59.91*
Central	1255	13	87	
Greater Accra	1677	6	94	
Volta	905	28	72	
Eastern	1520	7	93	
Ashanti	3101	3	97	
Brong-Ahafo	1138	13	87	
Northern	1086	44	56	
Upper East	359	15	85	
Upper West	265	24	76	
*Disability status*				
Has functional difficulty	974	22	78	33.69*
Has no functional difficulty	9887	13	87	
*Frequency of reading newspaper*				
Not at all	11,000	14	86	5.39*
Less than once a week	796	3	97	
At least once a week	559	3	97	
Almost every week	166	2	98	
*Frequency of listening to a radio*				
Not at all	3918	18	82	23.32*
Less than once a week	2213	12	88	
At least once a week	2672	10	90	
Almost every week	3779	10	90	
*Frequency of watching television*				
Not at all	3173	24	76	80.68*
Less than once a week	1478	17	83	
At least once a week	2187	11	89	
Almost every week	5744	6	94	

^*^p Value < 0.05

### Predictors of use of reusable menstrual materials among women of reproductive age

In the unadjusted analysis, all participant characteristics and exposure to mass media were statistically significant predictors of using reusable menstrual materials. For instance, women aged 45–49 years (COR = 6.13; 95% CI 4.66–8.06) were six times more likely to manage menses with reusable materials compared with women aged 15–19 years. Also, women who had at least primary education (COR = 0.31; 95% CI 0.24–0.39) were 69% less likely to use reusable materials compared with those with pre-primary or no education (Table 3). In the adjusted analysis (Table 3), women who were between the ages of 45–49 years (AOR = 5.34; 95% CI 3.47–8.19) were 5 times more likely to manage menstruation with reusable materials compared with those aged 15–19 years. Also, women who had attained at least primary school education (AOR = 0.50; 95% CI 0.38–0.66) were 50% less likely to use reusable materials compared with women who had pre-primary/no education. Also, wealth index was a significant predictor of using reusable materials. For instance, women classified in the middle wealth quintile (AOR = 0.66; 95% CI 0.50–0.88) were 34% less likely to use reusable materials to collect menstrual blood compared with women in the poorest wealth quintile. Further, women who had never married were less likely to use reusable materials compared with those who were currently married (AOR = 0.70; 95% CI 0.52–0.93). Women who were exposed to mass media had less odds of using reusable materials compared with women who were not exposed to mass media. Also, women with disabilities (AOR = 1.36; 95% CI 1.06–1.76) had higher odds of managing menstruation with reusable materials compared with their counterparts. The goodness of fit test (Hosmer–Lemeshow Test) showed that the model was well fitted (*p* = 0.78) (Table 3).

**Table 3:** Binary Logistic Regression analysis of predictors of usage of reusable menstrual materials

Characteristic	Crude analysis OR (95% CI)	Adjusted analysis OR (95% CI)
*Age groups (years)*		
15–19	1(ref)	1(ref)
20–24	0.92(0.68–1.26)	1.05(0.72–1.52)
25–29	1.58(1.18–2.12)*	1.45(0.95–2.20)
30–34	2.16(1.63–2.87)*	2.08(1.35–3.21)*
35–39	3.22(2.44–4.26)*	2.39(1.57–3.65)*
40–44	4.76(3.67–6.19)*	4.35(2.82–6.72)*
45–49	6.13(4.66–8.06)*	5.34(3.47–8.19)*
*Education levels completed*		
Pre-primary or no education	1 (ref)	1(ref)
Primary	0.31(0.24–0.39)*	0.50(0.38–0.66)*
Junior high	0.12(0.10–0.15)*	0.33(0.25–0.43)*
Senior high	0.04(0.03–0.06)*	0.24(0.16–0.34)*
Higher	0.02(0.01–0.05)*	0.18(0.09–0.35)*
*Wealth index*		
Poorest	1 (ref)	1(ref)
Second	0.65(0.51–0.84)*	1.01(0.77–1.32)
Middle	0.35(0.27–0.45)*	0.66(0.50–0.88)*
Fourth	0.13(0.10–0.18)*	0.32(0.23–0.44)*
Richest	0.06(0.04–0.09)*	0.19(0.12–0.29)*
*Marital status*		
Currently married	1 (ref)	1(ref)
Formerly married	0.79(0.63–1.00)*	0.86(0.69–1.08)
Never married	0.24(0.19–0.30)*	0.70(0.52–0.93)*
*Area of residence*		
Urban	1 (ref)	1(ref)
Rural	2.76(2.23–3.42)*	1.05(0.83–1.33)
*Region*		
Greater Accra	1 (ref)	1(ref)
Western	2.22(1.47–3.36)*	1.21(0.74–1.98)
Central	2.36(1.54–3.63)*	1.38(0.84–2.28)
Volta	6.08(3.85–9.60)*	2.58(1.62–4.11)*
Eastern	1.20(0.75–1.92)	0.52(0.30–0.88)*
Ashanti	0.41(0.24–0.70)*	0.19(0.11–0.32)*
Brong-Ahafo	2.23(1.47–3.37)*	0.88(0.53–1.45)
Northern	11.86(7.85–17.90)*	2.98(1.82–4.87)*
Upper East	2.62(1.70–4.03)*	0.56(0.32–0.99)*
Upper West	4.82(3.27–7.09)*	1.07(0.65–1.77)
*Disability status*		
Has no functional difficulty	1 (ref)	1(ref)
Has functional difficulty	1.84(1.49–2.27)*	1.36(1.06–1.76)*
*Frequency of reading newspaper*		
Not at all	1 (ref)	1(ref)
Less than once a week	0.21(0.12–0.36)*	1.02(0.58–1.79)
At least once a week	0.20(0.10–0.37)*	1.03(0.48–2.20)
Almost every week	0.05(0.00–0.40)*	0.43(0.06–2.97)
*Frequency of listening to a radio*		
Not at all	1 (ref)	1(ref)
Less than once a week	0.67(0.53–0.83)*	1.00(0.79–1.27)
At least once a week	0.47(0.38–0.59)*	0.97(0.76–1.24)
Almost every week	0.50(0.41–0.61)*	1.00(0.81–1.25)
*Frequency of watching television*		
Not at all	1 (ref)	1(ref)
Less than once a week	0.67(0.52–0.87)*	1.18(0.89–1.57)
At least once a week	0.40(0.32–0.51)*	0.86(0.65–1.13)
Almost every week	0.21(0.17–0.26)*	0.78(0.61–0.99)*
Population size: 10,860.982 Number of observations: 10,861 Number of strata: 20 Number of primary sampling units: 660 Design df: 640 F (36, 605): 21.87 Prob: < 0.0001 McKelvey and Zavoina's R-sqaure: 0.40		

*CI* confidence interval, *OR* odd ratio

*p Value < 0.05; ref (reference category)

## Discussion

The prevalence of reusable menstrual management materials was 13%. This implies that most Ghanaian women of reproductive age use disposable menstrual hygiene management materials. Although Ghana has no established benchmarks regarding reusable menstrual materials, this prevalence is commendable when juxtaposed with the findings of previous studies. For instance, prior studies revealed that 34–54% of adolescent girls in northern Ghana use reusable cloth to manage menstruation. Notwithstanding, the prevalence found in this study is higher than the prevalence of reusable menstrual materials among university students in Ghana (9.2%) [25]. The disparities in the findings may be attributed to differences in study locations and populations. This study analysed nationally representative data among women of reproductive age, while prior studies focused on specific geographical locations or social class.

In addition, existing studies elsewhere have reported higher prevalence of reusable menstrual materials. For instance, a study in Nepal revealed that 66.7% and 76.1% of women used reusable sanitary cloth before and after an earthquake respectively [26]. In India, the prevalence of reusable menstrual materials ranged from 42 to 51.2% among women of reproductive age [27–29]. On the contrary, a study in Kolkata, India reported that 6.5% of women in reproductive age use reusable menstrual materials [30]. Also, studies have reported that 4.8% and 1.4% of high school girls in Ethiopia and Benin use reusable menstrual materials respectively [31, 32]. To the best of the authors’ knowledge, this is the maiden study to report a prevalence of 13% for reusable menstrual materials among women of reproductive age. The differences in these findings may be attributed to differences in contextual and socio-demographic factors. Evidence shows that decisions on the type of menstrual materials to use are influenced by availability, cost and cultural beliefs surrounding menstrual materials [20, 21]. Also, young women(10–24 years) have negative attitudes toward reusable menstrual materials [9].

Age was a significant predictor of using reusable menstrual materials. This finding is supported by a previous study in India, where adult women preferred reusable materials and young women preferred disposable sanitary products [33]. Adolescent girls in Malawi perceived reusable materials as ‘old fashion’, hence preferred single-use pads [9]. A plausible explanation is that adult women may be more familiar with reusable pads compared with disposable pads [33]. In addition, women in rural areas were more likely to use reusable menstrual materials compared with those in urban settings. Prior studies have shown that women in remote areas preferred reusable pads, while those in urban areas preferred commercial sanitary pads [5]. Women in rural communities are more likely to be unemployed or engaged in menial jobs, hence may have little disposable income to buy commercial sanitary pads. Besides cost, disposable pads may not always be available in rural areas compared to urban areas. There is evidence to show that menstruators prefer reusable pads because they are cost-effective and available [7, 9].

Further, it was revealed in this study that women who were currently married or in a union had higher odds of using reusable materials compared with those who were never married or in a union. This finding is not surprising because never-married women are more likely to be adolescent or young women, while married women are more likely to be adult women. As indicated earlier, adult women prefer reusable menstrual materials to disposable pads [33]. We also found that less educated women were more likely to use reusable menstrual materials compared with highly educated women. This finding is expected because well educated women are more likely to be employed and can therefore afford disposable pads. Moreover, educated women are literate and can access health information regarding sanitary products. The use of reusable menstrual materials was common among women in the poor wealth index. Understandably, women of low economic status may not be able to afford commercial sanitary pads. Previous studies in Ghana have reported that financial constraint was a major barrier to accessing disposable sanitary products among adolescent girls in school [17, 18].

It was also revealed that women with disabilities preferred reusable materials compared with those without disabilities. Women with disabilities are less likely to be employed and may not be able to afford disposable pads every month. Women who were exposed to mass media had less odds of using reusable menstrual materials. In Ghana, commercial sanitary products are advertised on television, radio and in newspapers. Therefore, women who are exposed to the mass media may be influenced by these advertisements. Also, women in the Northern and Volta Regions were two times more likely to use reusable materials compared with women in the Western Region. This finding may be explained by the high incidence of poverty in the Northern and Volta regions compared with the Western Region [34]. Therefore, women from these geographical regions may not be able to afford commercial sanitary pads compared with their counterparts.

### Implications for policy, practice and research

These findings have implications for menstrual health policy, programming and research.

Reusable materials can breed infections when they are not well cleaned and stored [12]. Since most menstruators in Ghana dry their reusable materials indoors or under the shade; instead of drying them in the sunlight [23], they may be at a higher risk of infections, including candidiasis, bacterial vaginosis and urogenital infections. These infections are risk factors for HIV, adverse pregnancy outcomes and Human Papillomavirus Infection [35]. Untreated infections can lead to prenatal infection, infecundity, toxic shock syndrome, ectopic pregnancy and low birth weight [36].

It is therefore crucial for the Ghana Health Services to educate the public about good menstrual hygiene practices. Stakeholders can leverage the mass media to disseminate menstrual health information. In addition, the Ghana Education Service should intensify efforts towards menstrual health education by providing skilled-based education regarding the management of reusable menstrual materials as well as educating girls about safe reusable menstrual materials. There is also a need for social and behavioral change communication programmes to help reduce the stigma and shame associated with drying reusable menstrual materials in the sunlight. These efforts will help reduce the unhygienic management of reusable materials and infections among users.

In addition, it is necessary to promote access to disposable sanitary pads since it is safe and easy to use [4]. Currently, in Ghana, disposable sanitary pads attracts an import tax of 20% and a value-added tax of 12.5% [37]. There has been little commitment on the part of the Government of Ghana regarding implementing policies that will make disposable sanitary pads affordable. Other Africa countries, including Kenya, Ethiopia and Tanzania, have either reduced or eliminated taxes on sanitary products to make it affordable [38]. This study reiterates that it is necessary for the Government of Ghana to reduce taxes on sanitary pads to make it affordable and accessible to women and girls.

This study sets the pace for future research on the subject matter to help inform policy and programmes. Currently, little is known about the types of reusable menstrual materials patronized by women in Ghana as well as the health status of women who manage period with reusable materials. Also, future studies should explore the acceptability of safer and easy to clean reusable menstrual materials, such as the menstrual cup. Moreover, there is a paucity of literature on the socio-cultural correlates of reusable menstrual materials and the experiences of women who use reusable menstrual materials.

### Limitations and strengths of the study

This is a primal study in Ghana which investigates menstrual hygiene behaviours among women of reproductive age using nationally representative data, hence the findings of this study can be generalized to the study population. Also, we employed appropriate statistical analysis, such as weighting to adjust for oversampling and under-sampling. Moreover, the data analysed in this study was from the most recent MICS. Another major strength of this study is the good response rate (99.8%). In addition, this study provides relevant empirical information on menstrual hygiene management practices of Ghanaian women of reproductive age. Stakeholders, including the Ministry of Health and Ministry of Gender, Children and Social Protection and non-governmental organizations, can leverage this information to inform reproductive health policies and programmes which would contribute to promoting the health and wellbeing of women and girls.

However, this study has some limitations, including the study design. The cross-sectional survey nature of the study cannot establish a causal relationship between the independent and dependent variables; hence the findings must be interpreted with caution. Also, we acknowledge that the prevalence of reusable menstrual management materials may be under-reported or over-reported. Further, data collection was based on self-report, hence there were possibilities of recall bias and social desirability bias. Also, the 2017/18 Ghana Multiple Indicator Cluster Survey did not collect data on the types of reusable menstrual materials used by women, management of reusable menstrual materials and health status of women who use reusable menstrual materials. This information is useful for menstrual health education and should be collected in future surveys.

## Conclusions

This study has demonstrated that a minority of Ghanaian women of reproductive age use reusable menstrual materials. Age, lower education, low economic status, little exposure to mass media, being married, residing in a rural area, and geographical region were the significant determinants of using reusable menstrual materials. The findings of this study provide relevant information for menstrual health policy and programming. Clean and safe reusable menstrual materials should be available for women to make their preferred option. Reusable menstrual materials should be accessible through outlets and information regarding use disseminated through mass media.

## Data Availability

The data used in this study is owned by UNICEF, therefore, the authors cannot share the data. Interested persons can contact UNICEF for the data (contact via accra@unicef.org). The authors confirm they did not have any special access or privileges to the data that other researchers would not have.

## Abbreviations

MICS: Multiple Indicator Cluster Survey
MMM: Menstrual management materials
UNICEF: United Nations International Children's Emergency Fund
CAPI: Computer assisted personal interviewing
